# The Probabilistic Niche Model Reveals the Niche Structure and Role of Body Size in a Complex Food Web

**DOI:** 10.1371/journal.pone.0012092

**Published:** 2010-08-09

**Authors:** Richard J. Williams, Ananthi Anandanadesan, Drew Purves

**Affiliations:** Microsoft Research, Cambridge, United Kingdom; University of Fribourg, Switzerland

## Abstract

The niche model has been widely used to model the structure of complex food webs, and yet the ecological meaning of the single niche dimension has not been explored. In the niche model, each species has three traits, niche position, diet position and feeding range. Here, a new probabilistic niche model, which allows the maximum likelihood set of trait values to be estimated for each species, is applied to the food web of the Benguela fishery. We also developed the allometric niche model, in which body size is used as the niche dimension. About 80% of the links in the empirical data are predicted by the probabilistic niche model, a significant improvement over recent models. As in the niche model, species are uniformly distributed on the niche axis. Feeding ranges are exponentially distributed, but diet positions are not uniformly distributed below the predator. Species traits are strongly correlated with body size, but the allometric niche model performs significantly worse than the probabilistic niche model. The best-fit parameter set provides a significantly better model of the structure of the Benguela food web than was previously available. The methodology allows the identification of a number of taxa that stand out as outliers either in the model's poor performance at predicting their predators or prey or in their parameter values. While important, body size alone does not explain the structure of the one-dimensional niche.

## Introduction

Understanding the diversity and distribution of interspecies interactions is a vital challenge for developing our understanding of complex ecosystems. Ecological networks depict the complex patterns of interactions between species and provide an important tool for studying the diversity and complexity of ecosystems [1]. Feeding interactions, the primary mechanism by which energy and resources are passed between organisms, are fundamental to the functioning of ecosystems, and so networks of feeding interactions, or food webs, have long been a central paradigm of ecological thought [2]. The simplest representation of a food web, in which both species and interactions between species are represented as present or absent from the system, ignores many details but captures the topological structure related to the energy transfer processes occurring in the system. These binary food webs provide a tractable representation of ecological complexity, and their structure has important consequences for many aspects of ecosystem function, including the relationship between network complexity and system stability [3], their robustness and resilience to species extinctions [4] and their resilience in the face of environmental change [5].

One of the fundamental challenges in studies of the structure of food webs is been determining whether there are topological patterns that are universal across different food webs and if these patterns exist, determining the common processes that structure different food webs and give rise to these universal patterns. A wide variety of approaches have been used to study the mechanisms giving rise to regularities in complex food webs. These include models coupling evolutionary and population time scale [6], [7], models of food web assembly [8], studies of the effects of body size on the persistence of species in food webs [9], and models of network topology including models grounded in mechanistic concepts such as foraging theory [10], [11], and the stochastic structural food web models that are the focus of this work.

Two important ideas were used in early food web studies to interpret patterns seen in network structure. First, the idea of the ecological niche [12], in which species consume resources which fall within a restricted volume of a multi-dimensional space of ecological trait values. Early food web studies [13], [14] showed that in many smaller networks, species can be ordered such that all diets fall into a contiguous interval on a single dimension, suggesting that niche space can often be collapsed to a single dimension. Second, the idea that species are ordered into a hierarchy, with predator species consuming only those prey that are at or below the predator's position in the hierarchy. This is the driving principle constraining species diets in the cascade model [15]. A one-dimensional niche with interval diets and slightly relaxed hierarchical ordering were combined in the simple yet successful food web niche model [16]. Together with the important choice of the distribution of diet widths [17], these ideas comprise the essential elements of the niche model. Several variants of the niche model have since been proposed [18], [19], [20], [21], but depending on the methods used to compare the model and the empirical data, their performance is not very different from that of the original niche model [18], [21].

While the niche model has provided a reasonably successful model for the structure of a range of food webs, there has been little work exploring the ecological meaning of the single niche dimension. Early work on understanding the role of body size in determining species' diets [22], [23] suggested that body size ordering, with species only consuming prey smaller than themselves, drives the hierarchical structure that is one of the key assumptions of the niche model. Some other traits that potentially play a role, such as gape, mobility and range and metabolic traits, are typically highly correlated with body size. This has led to frequent speculation [11], [16], [18], [24], [25] that the niche axis is closely or directly related to body size. Recently, several studies have highlighted patterns in the structure of empirical food webs that are strongly related to the body sizes of species [25], [26]. The importance of body size has also been highlighted in several studies which show that populations in size structured food webs are more likely to be stable or persistent [9], [27], [28]. While a recent model based on the niche model explicitly assumes that species are ordered by their body size [11], a relationship between body size and niche model parameters has not yet been formally demonstrated, and the extent to which body size alone or in combination with other species traits determines food web structure is not yet well understood. Given the success of the niche model and its variants, determining which traits underlie the niche axis in the family of single dimensional niche-structured food web models remains a critical open question. Both the placement of species on the niche axis and the rules determining the width and placement of diets on the axis need to be better understood.

In part, the lack of evidence about the relationship between species' niche parameters and their biology (whether body size or some other aspect) reflects the way in which the niche model has been applied. To date, the niche model has usually been employed using what might be called a forward modelling approach: (i) the model structure is assigned; (ii) species are assigned parameters randomly from arbitrarily -assigned distributions; (iii) the resultant model is used to generate artificial food webs; (iv) aggregate features of the artificial webs are compared to data. Although this approach has proved useful, it prevents the detailed species-by-species analysis that is needed to uncover the biology underlying species' parameters. In contrast, in this study we use an *inverse* modelling approach: (i) the model structure is assigned; (ii) this structure is formally confronted with data using likelihood-based statistics; (iii) the result is a set of estimated niche model parameters for every species, which together describe a distribution, across all species, of each niche model parameter; (iv) the parameters can then be compared, species-by-species, with aspects of biology, and the distributions of the parameters can be compared with previous assumptions about these distributions.

To enable this inverse approach, we developed a simple probabilistic variant of the niche model. This model, like the original niche model, has a single niche dimension and three parameters associated with each species: the species' position on the niche axis (niche position), the position of its diet on the niche axis (diet position) and the width of its diet on the feeding axis (feeding range). Using standard statistical techniques, we fit the probabilistic niche model to a widely studied empirical data set known to be reasonably well-described by the niche model, and which has estimates of body sizes for all taxa. We then examine the best-fit (MLE) parameter values of the model to better understand the reasons for the successes and failures of the niche model, and to interpret the meaning of the various species parameters, particularly in how they relate to body sizes in the food web. We also explore where model predictions are good or where there is a large mismatch between model and data on a species-by-species basis. This approach allows us to perform a much more detailed comparison between an observed food web and a stochastic food web model than has previously been performed.

## Methods

### Probabilistic Niche Model

**Figure 1:**
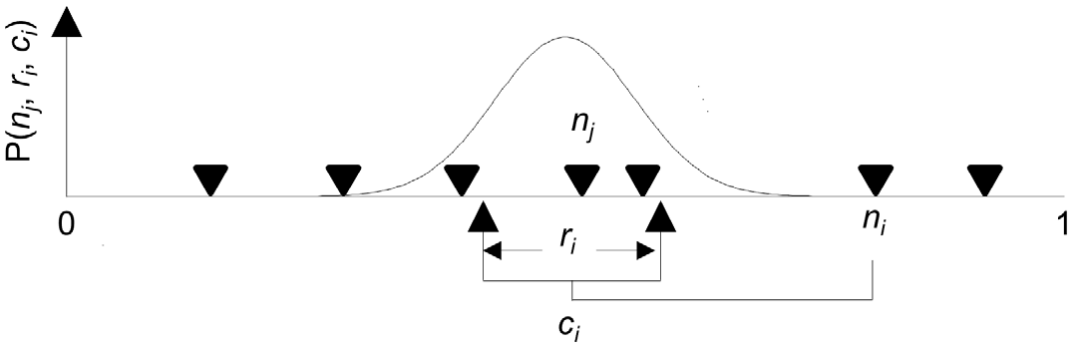
Diagram of original and probabilistic niche models. Diagram of the original niche model, in which species *i* consumes all species within the range *r_i_*, and the probabilistic niche model, in which the probability that species *i* consumes species *j* is defined by the probability P(*n_j_*, *r_i_*, *c_i_*).

A binary food web with *S* species and *L* links can be represented as an *S*×*S* connection matrix where entry *i*,*j* represents a possible link in the food web and is either 1 (species *i* eats species *j*) or 0 (species *i* does not eat species *j*). The original formulation of the niche model (Williams and Martinez 2000) makes a prediction for each link *i*,*j* in the food web of either 1 or 0, depending on whether the prey species *j* lies within the feeding range of the predator species *i* (Fig. 1). This formulation of the model cannot readily be employed within a likelihood-based context for three reasons. First, the formulation is only probabilistic when an ensemble of parameter values is considered, i.e., for a particular parameter set it predicts that *i* eats *j* or does not with certainty. Second, some links cannot be reproduced by the niche model (their probability is zero) [18], whereas likelihood-based statistical methods require that, for any parameter set, the model returns a *non-zero probability* that *i* eats *j*, for any link *i*, *j* (see eq. 2 below). Third, under the original formulation, the predictions of the model are discontinuous against the parameters. That is, the prediction for a given link *i*, *j*, can go through a sudden qualitative change (1 to 0, or 0 to 1) from an infinitesimal quantitative change in the value of one or more parameters. This occurs, for example, when the feeding range of *i* is increased just enough to include the niche position of *j*. Such discontinuities make parameter estimation hard in practise.

We made minimal changes to the Williams and Martinez (2000) formulation of the niche model to facilitate likelihood-based analysis (Fig. 1). We used a Gaussian formulation for the probability that species *i* eats species *j*:
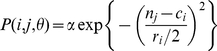
(1)where 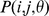 is the probability that species *i* eats species *j* given a particular parameter set 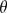 where 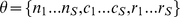; the parameter 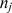 is the niche position of species *j*; the parameter 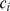 is the optimal diet position of species *i*; the parameter 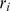 is the feeding range of species *i* ; and the parameter 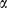 is the probability that *i* eats *j*, when *j* is exactly on *i*'s feeding optimum (i.e. when 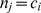). In principle, any unimodal function could be used in place of the Gaussian.

Under this formulation: (i) there is always a non-zero probability that any species *i* eats any species *j*; (ii) this probability is higher when 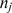 is close to 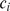; (iii) the rate that the probability declines as 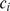 gets further from 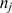, is set by the feeding range 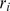; (iv) while the model imposes niche structure, there are no constraints on *c_i_*, so the hierarchical structure of the niche model is not imposed. In principle, the parameter 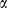 could take any value between 0 and 1 and could also vary from species to species; however, in the spirit of the original niche model we set 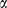 to a value very close to 1.0 (we used 0.9999 – a value of exactly 1.0 would have caused numerical errors) for all species. When 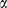 was included as a free parameter (results not shown), its estimated value was very close to 1.0 anyway, and the qualitative conclusions were no different from those presented here.

Given the evidence suggesting that diets are strongly controlled by the relative body sizes of predators and prey, we created a version of the probabilistic niche model that we call the *allometric niche model*, in which niche positions *n_i_* are not free parameters, but instead are functions of species' body masses. To constrain *n_i_* to range from 0 to 1, we set 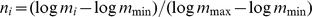 where *m_i_* is the body mass of species *i* and 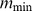 and 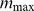 are the minimum and maximum of these values observed within the entire set of species. The parameters *r_i_* and *c_i_* remain free parameters as in the probabilistic niche model, and so the allometric niche model has the parameter set 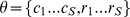.

We find the maximum likelihood set of parameters for the probabilistic and allometric niche models given the observed feeding relationships in the data. The set of model parameter values for a network with *S* species is given by 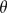, while 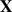 is the data, i.e., 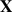 is an *S*×*S* connection matrix containing an observation 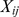 for each link *i*, *j* (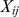 = 1 means *i* eats *j*; 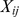 = 0 means *i* does not eat *j*). We use simulated annealing [29] to find the maximum likelihood parameter set where the log-likelihood is defined as:
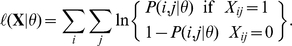
(2)The end results of the analysis of the model are: (i) a single vector 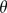 that gives the best fit to the data referred to as the maximum likelihood (MLE) parameter estimates; (ii) a set of model predictions (evaluated at the MLE) to compare with observations; (iii) a measure of overall goodness-of-fit including a penalty for extra parameters (AIC) [30] with which to select between different models. This basic methodology, also applied in other recent food web studies [18], [31], is widely used in other areas of ecology [32].

As a simple measure of goodness of fit comparable with previous work, we calculated the expected fraction of observed links (i.e. those links *i*, *j* where 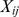 = 1 in the connection matrix) correctly predicted by the model when realized at the MLE. The expected number of links is very close to the observed number of links so this serves as an easily understood measure of the overall performance of the model [11]. Note that if the total number of links predicted by the model is significantly different from the total number observed, this is not a useful measure of model performance – for example a naive model that predicts every link is present always predicts every observed link correctly, but at the expense of also incorrectly predicting every non-existent link. The expected number of correctly predicted links is defined as: 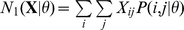 and the expected fraction of links predicted correctly is *f_L_* = *N*_1_/*L*. We also computed the fraction of links correct for each row and column in the connection matrix in order to compute the fractions of each species' predators and prey correctly predicted. The expected number of prey (resource) links is 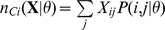 while the expected number of predator (consumer) links is 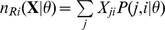. Then the fractions of predator and prey links correctly predicted are *f_Ri_* = *n_Ri_*/*R_i_* and *f*_Ci_ = *n_Ci_*/*C_i_* respectively, where *R_i_* and *C_i_* are the number of resources and consumers of species *i*.

The values of *c* and *r* for primary producers are fixed at *c* = 0 and *r*→0 rather than being free parameters. This forces all their link probabilities to be very small. Similarly, the *r* of species that consume a single prey are fixed – the link probabilities of a specialist will closely follow the empirical data as long as *c* is equal to the *n* of its prey and *r*→0.

### Data

The study was conducted using the Benguela food web [33], a pelagic marine food web with S = 29 taxa, which in this food web typically represent groups of functionally similar organisms. There are L = 203 links, therefore *L/S* = 7.0 links per species and directed connectance *C = L/S* ^2^ = 0.24. This food web has been widely used in other food web model studies [11], [16], [18], [20], [24] as its structure is known to be reasonably well-predicted by the niche model and its variants, and estimates of average body mass are available for all taxa [33]. Nevertheless, it suffers from some of the problems typical of food web data [34], in particular uneven taxonomic aggregation, with taxa quite finely resolved among the fish, but much more coarsely resolved among other organisms.

## Results

### Fit to observations

**Figure 2:**
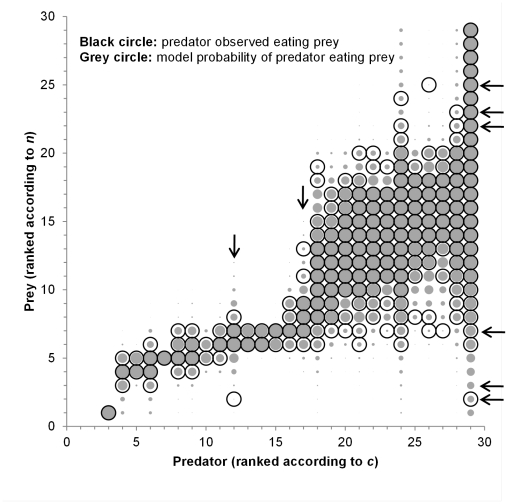
Probabilistic niche model results for the Benguela food web. Feeding links in the empirical data set and feeding probabilities in the probabilistic niche model for the maximum likelihood estimate (MLE) parameter set. On the x-axis, predators are ordered by their estimated (MLE) *c_i_* values; on the y-axis, prey are ordered by their estimated (MLE) *n_i_* values. Model predictions, calculated at the MLE, are shown as the grey circles: the area of each circle is proportional to P(*n_j_*, *r_i_*, *c_i_*), the probability that *i* eats *j*. Apparent missing grey circles simply correspond to very low values of P(*n_j_*, *r_i_*, *c_i_*). Observations are shown in black: a black circle is shown for those feeding relationships that have been observed. A match between large grey circles, and the black circles, implies a close match between model and data. Two predators with poorly predicted prey (expected fraction of prey links ≤0.65), *other pelagic* and *chub mackerel*, are labelled with arrows. Six prey species with poorly predicted predators (expected fraction of predator links ≤0.65) are labelled with arrows: from bottom to top, *gelatinous zooplankton*, *bacteria*, *macrozooplankton*, *snoek*, *sharks* and *kob*.

On a link-by-link basis, the model reproduced the food web topology quite well (Fig. 2). The expected total number of links produced by the MLE parameter set of the probabilistic niche model is 197.0, 97% of the 203 links in the empirical data set. On average, the probabilistic niche model reproduced 79.7% of the observed links and 90.6% of the connection matrix entries (0 or 1) correctly. In contrast, a random model constrained to have the same connectance as the empirical data would reproduce a fraction *C* (24%) of the links and 1−2*C*−*C* ^2^ (63.4%) of the connection matrix entries correctly. The maximum log-likelihood of the probabilistic niche model 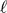 = −105, and its AIC = 385, while the maximum log-likelihood of the random model 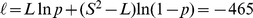 when *p* = *L/S* ^2^, and its AIC = 932. The log-likelihood of the minimum potential niche model [18], the best-performing model to date, is 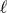 = −214, and the model has *S*+3 parameters, giving AIC = 493 (results are summarized in table 1).

**Table 1:** Comparative performance of models.

Model	Params	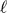	AIC	Links	Entries
PNM	87	−105	385	0.797	0.906
*n*, *c* free; *r* = *f*(*c*)	60	−141	402	0.734	0.883
*n*, *c* free; *r* = *f*(*n*)	60	−151	421	0.734	0.880
*n*, *r* free, *c* = *f*(*n*)	60	−174	469	0.754	0.875
*n* free, *c* = *f*(*n*), *r* = *g*(*n*)	33	−205	475	0.712	0.855
ANM	58	−209	535	0.681	0.845
MPNM	32	−214	493		

Params is the number of model parameters; 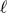 is maximum log-likelihood; Links is the fraction of links reproduced by the model; Entries is the expected fraction of connection matrix entries reproduced by the model. PNM is probabilistic niche model with *n_i_*, *c_i_* and *r_i_* all free parameters. ANM is the allometric niche model. MPNM is the minimum potential niche model [18]. Other models are variants of the PNM with one or two parameters a linear function of another parameter.

**Figure 3:**
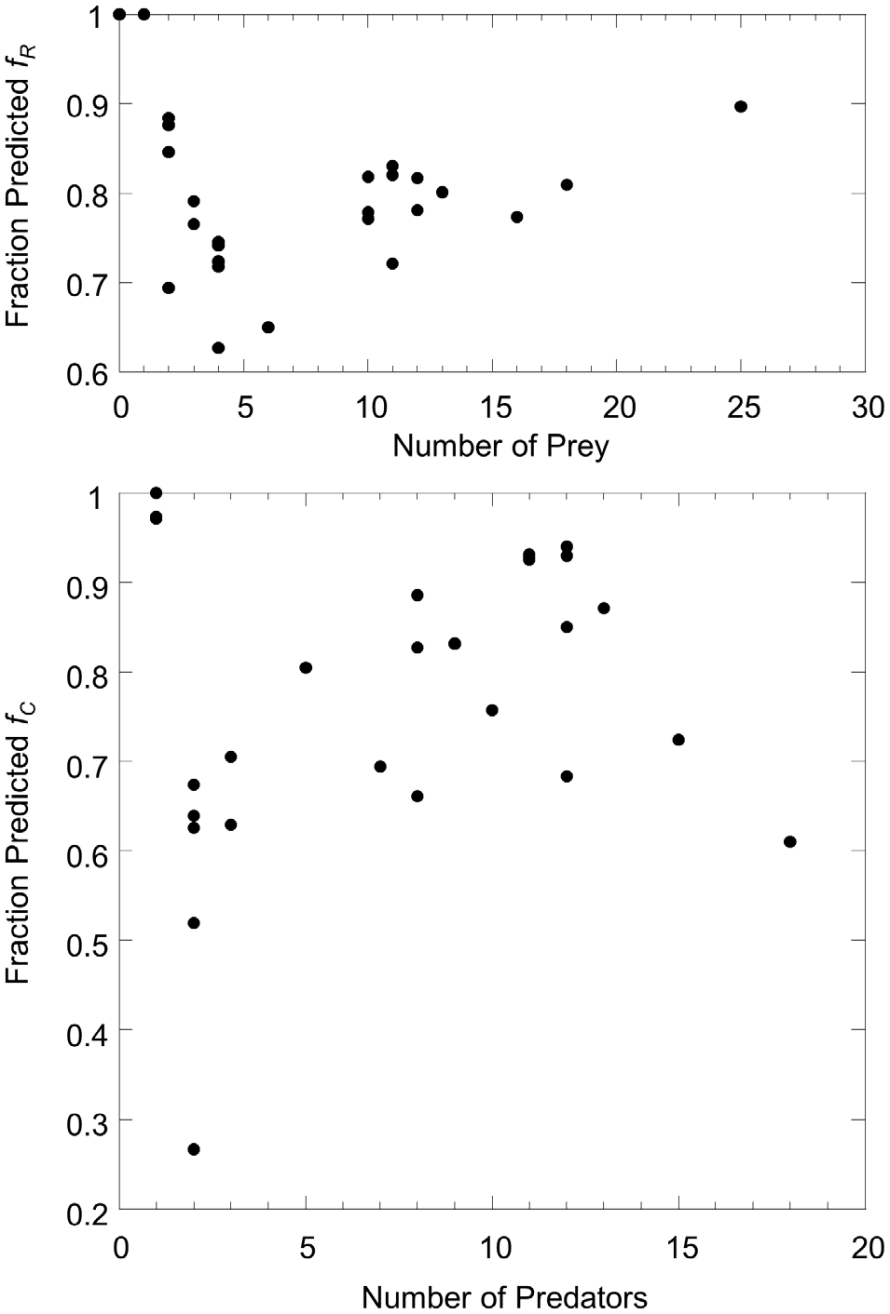
Fraction of links reproduced correctly for each species. (a) Number of prey versus *f_R_*, the expected fraction of prey links reproduced correctly and (b) Number of predators versus *f_C_*, the expected fraction of predator links reproduced correctly.

Visual comparison of predictions versus observations (Fig. 2) and the species-by-species fractions of links correctly reproduced (Fig. 3) reveals that model-data mismatch is unevenly distributed across the connection matrix. The model falls short in its representation of the diets of two specialist species (*f_R_* ≤0.65 for *other pelagic* and *chub mackerel*), and the fraction of predators of several relatively invulnerable species (*f_C_* ≤0.65 for *gelatinous zooplankton*, *kob*, *bacteria*, *snoek*, and *sharks*) and one highly vulnerable species (*macrozooplankton*). Prediction of the predators of gelatinous zooplankton is particularly poor, with *f_C_* = 0.27. What these model-data mismatches share is the non-intervality of the predator's diets. That is, because of its structure, the niche model is not able to reproduce the diets of predators that consume non-interval sets of prey. The model, however, does show that the non-intervality of predators' diets usually occurs toward the edges of their feeding ranges, suggesting that predators with non-interval diets still tend to have a ‘core’ interval diet composed of prey with *n_j_* values closer to the predator's *c_i_* value (Fig. 2).

### Correlations among parameters

**Figure 4:**
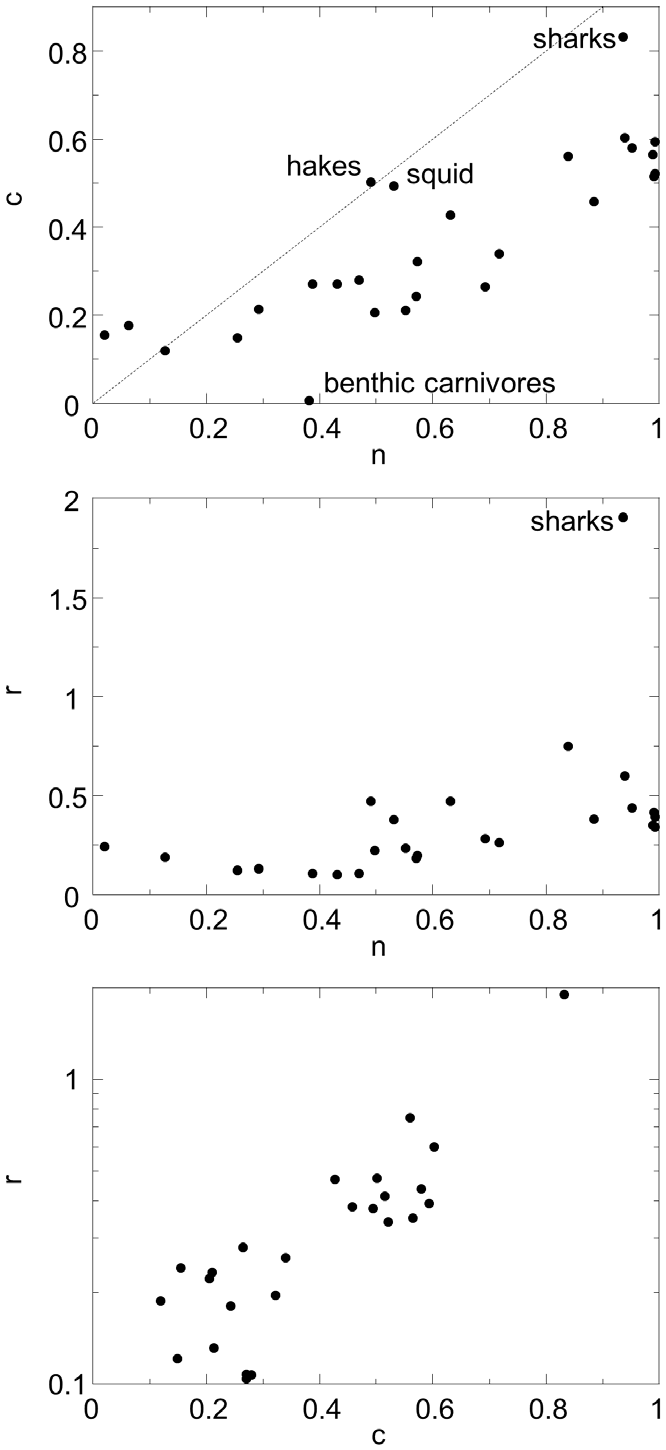
Relationships between maximum likelihood parameters. (a) Feeding range *r_i_* and (b) centre of feeding range *c_i_* versus niche position *n_i_* and (c) *r_i_* versus *c_i_* for the MLE parameter set of the probabilistic niche model.

Analysis of parameter values reveals that *n*, *c*, and *r* are positively correlated (Table 2), with the exception of a few outliers seen in scatterplots of variable pairs (figures 4). Outliers include benthic carnivores, hake, squid and sharks in the *n* vs. *c* plot (Fig 4a) and sharks in the *n* vs. *r* plot (Fig. 4b). Figure 4a shows that the model is hierarchically structured, with almost all *c_i_*<*n_i_*, while figure 4c shows that there is an exponential relationship between *c* and *r*.

**Table 2:** MLE parameter Spearman rank correlations and p values.

Parameters and variables	Correlation	p
*n*, *c*	0.872*	6.89×10^−10^
*n*, *r*	0.755*	2.17×10^−6^
*c*, *r*	0.843*	9.35×10^−9^
*n*, body mass	0.883*	2.23×10^−10^
*c*, body mass	0.862*	1.80×10^−9^
*r*, body mass	0.826*	3.48×10^−8^
*n*, *x*	0.166	0.388
*x*, body mass	0.288	0.130
*n*, *c′*	−0.137	0.477
*c′*, body mass	−0.000739	0.997

Entries marked with * have significant correlation (p<0.001) while all other entries have *p*>0.05 when corrected using false discovery rate control ([36]).

We found no significant correlation between the *ratio x* = *r*/*n*, and *n*; or the *ratio c′* = *c*/*n*, and *n* (Table 2). This suggests that the strong correlations among *n*, *c* and *r* result primarily from both the feeding range *r* and feeding optimum *c* scaling linearly with niche position *n*. Both features were included as *a priori* assumptions in the original niche model, but have been extracted from the data set studied here by the inverse approach.

The correlations between the parameter pairs suggest a large amount of redundancy in the observed web; that is, species occupy only a subset of the possible parameter combinations, such that much of the food web structure would be retained by a model with fewer parameters. For example, we found that 80% of the interspecific variation in parameters was captured by the first PCA axis (details of PCA analysis not shown), suggesting that in principle a model allowing only one free parameter per species would retain most of the food web structure. We implemented a family of model variants in which one or both of *c_i_* and *r_i_* are functions of *n_i_*, or *r_i_* is a function of *c_i_*, leading to a significant reduction in the number of model parameters. Linear, exponential and power of functional forms were tried, with the best results when a linear relationship was used for *r* and *c* vs. *n*, 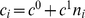 and/or 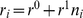, and exponential for *r* vs. *c*, 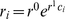, where *c^0^*, *c^1^*, *r^0^* and *r^1^* are free parameters. This is not surprising given the relationships apparent in figure 4. Results are given in Table 1. The models generally performed quite well: all four restricted-parameter models outperformed the minimum potential niche model, and the model where *r_i_* is an exponential function of *c_i_* slightly outperformed the fully parameterized model.

### Distributions of parameters

The original niche model assumes certain distributions for *n*, *x* and *c*. Here we test whether the distributions of the parameters of the probabilistic niche model follow those assumed by the niche model. In the original niche model, species' niche positions are assumed to be uniformly distributed between 0 and 1; the distribution of n of the probabilistic niche model is well-explained by a uniform distribution (K-S test, p = 0.18). The original niche model sets *r_i_* = *x_i_n_i_* where *x_i_* is drawn from a beta distribution with a mean of 2*C*. In the probabilistic niche model, the upper limit of *r* is not constrained and the distribution of *r_i_* is well-explained by an exponential distribution (distribution scale *β* = 0.73, K-S test *p* = 0.31). An exponential distribution of *x* in the original niche model has previously been shown to be vital for reproducing many features of empirical food webs [17]. The original niche model also constrains *c_i_* to values less than *n_i_*, and draws *c_i_* from a uniform distribution across its range of possible values. We therefore tested the distribution of *c*′, and after excluding the three species with *c′*>1, (gelatinous zooplankton, bacteria and hake), found that the distribution of *c′* is not well-explained by a uniform distribution (K-S test *p* = 0.004).

### Body mass

**Figure 5:**
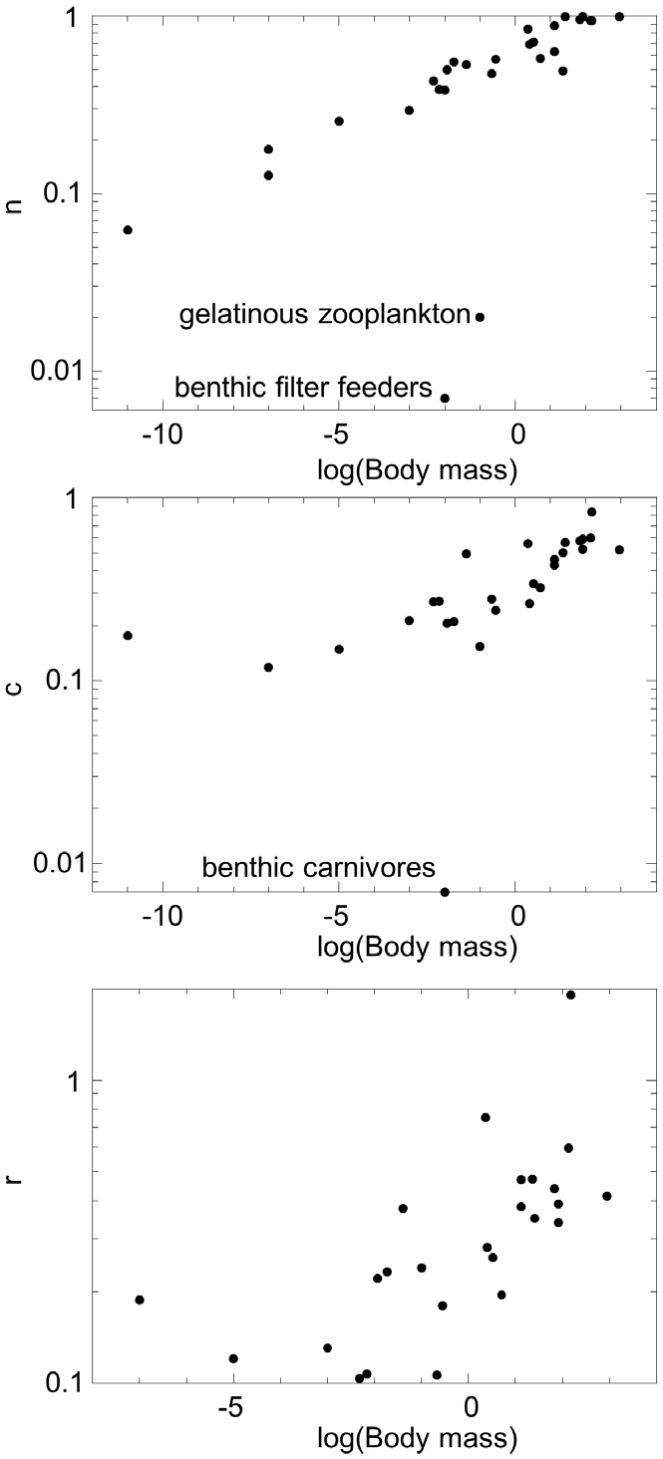
Relationships between maximum likelihood parameters and body size. (a) Niche position *n_i_* (b) feeding range *r_i_*, and (c) centre of feeding range *c_i_* versus log(body mass) for the for the MLE parameter set of the probabilistic niche model.

All three parameters were positively correlated with body mass (Fig 5, Table 2), such that larger species tend to have higher *n*, higher *c*, and higher *r* values. Exceptions to this pattern (Fig 5) include gelatinous zooplankton and benthic filter feeders for *n* vs. body mass, and benthic carnivores for *c* vs. body mass. A log-log plot (figure 5a) clearly shows that apart from the two outliers, the relationship between *n* and body mass closely follows a power law.

The strong correlations between the parameters and body mass motivated the development of the allometric niche model, which successfully predicted 68% of the links in the network and had AIC = 535 compared to AIC = 385 for the probabilistic niche model. The lower AIC of the probabilistic niche model shows that the added freedom in *n_i_* values in this model significantly enhances its ability to reproduce the empirical food web studied here compared to the allometric niche model, which has niche position equal to log of body mass.

### Discussion

The overall fit of the probabilistic niche model to the Benguela food web is significantly better than that of any of the models tested in two recent studies that computed the likelihoods of various food web models, including the best-performing model to date [18], [31]. This improved performance occurs because the way in which the probabilistic niche model allows gaps in the exactly interval diets of the original niche model more closely mirrors the niche structure of the empirical data than the non-interval niches used in the minimum potential niche model or other niche model variants. In particular, the probabilistic niche model produces niches that are high probability and therefore highly contiguous in the centre of the niche and low probability and therefore more fragmented toward their margins, rather than being of uniformly lower probability throughout their range [18], [21]. In addition, outside the high-probability centre of the niche, feeding probabilities in the probabilistic niche model decline continuously with distance from the feeding range centre. This is unlike feeding probabilities in the generalized niche model [20] or in a niche model with randomly placed non-interval links [18], which remain constant even for species far from a predator's high-probability niche centre.

The original niche model places three important constraints on species diets – (1) they lie on a single-dimensional niche; (2) they fall in a contiguous range of that niche dimension and (3) species are hierarchically ordered, so there is an arrangement of species where all diet centres fall below their position on the niche axis. The model has also assumed specific probability distributions for feeding ranges and for diet positions on the niche axis. The probabilistic niche model similarly assumes one dimensional, near-contiguous diets, and the best fit model parameters nearly have the hierarchical structure of the niche model, with only the three lowest-*n* species having *c*>*n*, and one higher-*n* species (hakes) having *c* slightly larger than *n*. The probabilistic niche model separates the assumptions of niche and hierarchy, rooted in ecological principles, from the assumptions of the probability distributions of the species' parameters. For the Benguela data set, the distribution of *n* is well-fitted by a uniform distribution and the distribution of *x* = *r/n* is well-explained by an exponential distribution [17] but the distribution of *c′* = *c/n* is not well-fitted by the uniform distribution assumed in the original niche model. In future studies, it will be interesting to test whether some data sets that are not well-explained by the original niche model are well-explained by the probabilistic niche model, and so are still constrained by niche and hierarchy but have trait distributions very different from those assumed in the original niche model.

The strong correlations between the parameters of the probabilistic niche model and species' body size (Fig. 5) and the relative success of the allometric niche model provide a biological explanation for the fact that the three parameters are so closely correlated among species (Fig 4) and for the hierarchical nature of the food web (Fig. 4 top). They show that in this food web, body size or other traits highly correlated with body size strongly constrain species' diets and that the frequent conjecture that the axis of the niche model maps onto body size is largely justified for this data set. Recent results [11] suggest that while body size plays an important role in determining the niche structure of some food webs, including the Benguela web studied here, it plays a much less important role in other food webs. In those food webs, we would not expect body size to be so strongly correlated with niche model parameters. It is likely that this hierarchical relationship occurs in part because the Benguela food web lacks parasites, which would break up the consistent pattern of large taxa consuming smaller taxa.

The significantly worse performance of the allometric niche model compared to the probabilistic niche model shows that body size, while very important in determining food web structure, is not the only species trait determining the structure of feeding niches. The non-interval nature of diets at the margins of their feeding ranges suggests that either a small number of additional trait dimensions [18], [35] or stochasticity (effectively very high dimensionality) is needed to capture species' diets more accurately.

The recent allometric diet breadth model (ADBM) [11] assumes that species lie on a one dimensional niche (as in the niche model) and that this niche dimension maps onto body size. For the Benguela food web, the best-performing version of the ADBM, with a ratio handling time function, successfully predicted 57% of the links in the food web. In contrast, the probabilistic niche model predicted 80% of the links and the allometric niche model predicted 68% of the links. Like the two models presented here, the ADBM assumes a hierarchically organized, single dimensional niche with near-contiguous diets. The ability of the probabilistic and allometric niche models to correctly represent a much larger fraction of links than the ADBM suggests that the various assumptions and scaling approximations used to determine foraging parameters in the ADBM are not optimal. In contrast, a best-fit ADBM, derived using the inverse approach employed here, could provide insight into the empirical relationship between body mass and foraging parameters.

The probabilistic niche model produces species-by-species estimates of parameters, which allows for a fine-grained analysis of the network. A number of taxa stand out as outliers either in the model's poor performance at predicting their predators or prey (figures 2 and 3) or in their best-fit model parameter values (figures 4 and 5). Species with parameter values that are outliers are not necessarily poorly predicted by the model, but parameter value outliers do make difficult the creation of accurate, less parameter-rich models in which one parameter is a simple function of other parameter values. The results for the less-parameterized models in table 2 show the importance to overall model performance of the outlier species. The *sharks*, *hake* and *squid* are outliers in the *n_i_* vs. *c_i_* plot (Fig 4a) and this lead to the worse performance of the model with *n_i_* and *r_i_* free and *c_i_* a linear function of *n_i_*. Similarly, *sharks* are outliers in the *n_i_* vs. *r_i_* plot (Fig. 4b) and this lead to the worse performance of the model with *n_i_* and *c_i_* free and *r_i_* a linear function of *n_i_*.

These outliers can be understood as occurring either due to their unique biology or limitations in the data. For example, *sharks* consistently stand out as exceptional consumers. They are highly general and so have an unusually broad feeding range *r*, placed relatively high on the niche axis (large *c*) (figure 4a and b). However, despite their unusual niche range and position relative to their location on the niche axis, their range and position fall within the exponential *c*-*r* relationship shown in figure 4c. Thus, while its parameter values are outliers, the *sharks*' diet, given those parameters, is reasonably well-predicted by the model. An important question for future studies is whether sharks stand out as outliers in terms of their parameter values or generality in other food webs.

The diet of *chub mackerel* is poorly predicted largely because there are gaps in its diet that are not present in the diets of other species (figure 2, *f_R_* = 0.65 in figure 3a). These gaps occur because, unlike several other predatory fish in this food web, it consumes *round herring* and *anchovy* but not *lightfish* and *hake*. This contrasts with the diet of the similar- sized *horse mackerel* (the taxon to the left in figure 2), which does not consume either *round herring* and *anchovy*. A check of online resources (fishbase) shows both mackerel listed as having similar diets, so it is not clear why they have different diets here, especially since taxa in this food web are generally broadly aggregated groups of organisms. It is beyond the scope of this work to further determine whether the poorly-determined diet of *round herring* is due to limitations in the data set or specific features of its biology.

The *benthic carnivores* taxon stands out by having a low niche position relative to its niche value and body size (figure 4a and 5b) and a narrow feeding range relative to its size (figure 5c). It is also an outlier in terms of its role in the food web. It only consumes the *filter feeder* taxon, which has a low niche value relative to its body size (figure 5a), and the *filter feeder* taxon is a basal species in this food web, with no diet specified, despite their role in the ecosystem as a consumer. The unusual niche values associated with both taxa likely occur in part because these taxa are particularly highly aggregated and have poorly resolved diets. Habitat heterogeneity is also potentially driving these taxa's niche values - they are the only benthic taxa in an otherwise pelagic food web. Other taxa that stand out as outliers are *gelantinous zooplankton* and *macrozooplankton*. *Gelatinous zooplankton* stands out as a taxon whose prey is poorly predicted by the model (figure 2 and 3); it also has a very low niche value *n* relative to its body mass (figure 5a). *Macrozooplankton* stands out as a taxon whose prey and predators are both poorly predicted by the model (figure 2 and 3). Of the relatively vulnerable taxa (those with number of predators greater than *L*/*S*), its predators are most poorly predicted by the model.

The probabilistic niche model, combined with inverse methods for comparing model and data, allows far more detailed comparisons between the model and the empirical data than has been possible before. The best-fit parameter set provides a significantly better model of the structure of the Benguela food web than previously available. Since parameters are estimated for each species, it is possible to identify specific species whose diets and consumers are well-predicted by the model and ones that are not as well-predicted, and connect those to details of the biology or idiosyncrasies of the data set. It is also possible to extract parameter distributions that best-fit the data, rather than assuming them a priori, as has been done in most previous structural food web models. This level of insight into food web structure is novel and allows the abstractions of the model and ecological details of empirical data to be drawn closer together than before.

## References

[1] Bascompte J (2009). Disentangling the web of life.. Science.

[2] Dunne JA, Pascual M, Dunne JA (2006). The network structure of food webs.. Ecological Networks: Linking Structure to Dynamics in Food Webs.

[3] May RM (1972). Will a large complex system be stable?. Nature.

[4] Dunne JA, Williams RJ, Martinez ND (2002). Network structure and biodiversity loss in food webs: robustness increases with connectance.. Ecology Letters.

[5] Petchey OL, McPhearson PT, Casey TM, Morin PJ (1999). Environmental warming alters food-web structure and ecosystem function.. Nature.

[6] Drossel B, McKane AJ, Quince C (2004). The impact of nonlinear functional responses on the long-term evolution of food web structure.. Journal of Theoretical Biology.

[7] Loeuille N, Loreau M (2005). Evolutionary emergence of size-structured food webs.. Proceedings of the National Academy of Science, USA.

[8] Morton RD, Law R, Pimm SL, Drake JA (1996). On models for assembling ecological communities.. Oikos.

[9] Brose U, Williams RJ, Martinez ND (2006). Allometric scaling enhances stability in complex food webs.. Ecology Letters.

[10] Beckerman AP, Petchey OL, Warren PH (2006). Foraging biology predicts food web complexity.. Proceedings of the National Academy of Science, USA.

[11] Petchey OL, Beckerman AP, Riede JO, Warren PH (2008). Size, foraging, and food web structure.. Proceedings of the National Academy of Science, USA.

[12] Hutchinson GE (1959). Homage to Santa Rosalia or Why are there so many kinds of animals?. American Naturalist.

[13] Cohen JE (1977). Food Webs and Dimensionality of Trophic Niche Space.. Proceedings of the National Academy of Sciences of the United States of America.

[14] Cohen JE (1978). Food Webs and Niche Space.

[15] Cohen JE, Briand F, Newman CM (1990). Community food webs: data and theory.

[16] Williams RJ, Martinez ND (2000). Simple rules yield complex food webs.. Nature.

[17] Stouffer DB, Camacho J, Guimera R, Ng CA, Amaral LAN (2005). Quantitative patterns in the structure of model and empirical food webs.. Ecology.

[18] Allesina S, Alonso D, Pascual M (2008). A General Model for Food Web Structure.. Science.

[19] Cattin M-F, Bersier L-F, Banasek-Richter C, Baltensperger R, Gabriel J-P (2004). Phylogenetic constraints and adaptation explain food-web structure.. Nature.

[20] Stouffer DB, Camacho J, Amaral LAN (2006). A robust measure of food web intervality.. Proceedings of the National Academy of Science, USA.

[21] Williams RJ, Martinez ND (2008). Success and its limits among structural models of complex food webs.. Journal of Animal Ecology.

[22] Cohen JE, Pimm SL, Yodzis P, Saldana J (1993). Body Sizes of Animal Predators and Animal Prey in Food Webs.. Journal of Animal Ecology.

[23] Warren PH, Lawton JH (1987). Invertebrate Predator-Prey Body Size Relationships - an Explanation for Upper-Triangular Food Webs and Patterns in Food Web Structure.. Oecologia.

[24] Stouffer DB, Camacho J, Jiang W, Amaral LAN (2007). Evidence for the existence of a robust pattern of prey selection in food webs.. Proceedings of the Royal Society of London Series B-Biological Sciences.

[25] Woodward G, Ebenman B, Emmerson M, Montoya JM, Olesen JM (2005). Body size in ecological networks.. Trends in Ecology & Evolution.

[26] Brose U, Jonsson T, Berlow EL, Warren P, Banasek-Richter C (2006). Consumer-resource body-size relationships in natural food webs.. Ecology.

[27] Otto SB, Rall BC, Brose U (2007). Allometric degree distributions facilitate food-web stability.. Nature.

[28] Emmerson MC, Raffaelli D (2004). Predator–prey body size, interaction strength and the stability of a real food web.. Journal of Animal Ecology.

[29] Kirkpatrick S, Gelatt CD, Vecchi MP (1983). Optimization by Simulated Annealing.. Science.

[30] Akaike H (1974). A new look at the statistical model identification.. IEEE Transactions on Automatic Control.

[31] Allesina S, Pascual M (2009). Food web models: a plea for groups.. Ecology Letters.

[32] Hilborn R, Mangel M (1997). The Ecological Detective: Confronting Models with Data.

[33] Yodzis P (1998). Local trophodynamics and the interaction of marine mammals and fisheries in the Benguela ecosystem.. Journal of Animal Ecology.

[34] Cohen JE, Beaver RA, Cousins SH, DeAngelis DL, Goldwasser L (1993). Improving food webs.. Ecology.

[35] Warren PH, Hochberg ME, Clobert J, Barbault R (1996). Structural constraints on food web assembly.. Aspects of the Genesis and Maintenance of Biological Diversity.

[36] Verhoeven KJF, Simonsen KL, McIntyre LM (2005). Implementing false discovery rate control: increasing your power.. Oikos.

